# Influence of Different Carrier Gases, Temperature, and Partial Pressure on Growth Dynamics of Ge and Si Nanowires

**DOI:** 10.3390/nano13212879

**Published:** 2023-10-30

**Authors:** Nicolas Forrer, Arianna Nigro, Gerard Gadea, Ilaria Zardo

**Affiliations:** 1 Department of Physics, University of Basel, Klingelbergstrasse 82, CH-4056 Basel, Switzerland; nicolas.forrer@unibas.ch (N.F.); arianna.nigro@unibas.ch (A.N.); gerard.gadea@unibas.ch (G.G.); 2 Swiss Nanoscience Institute, University of Basel, Klingelbergstrasse 82, CH-4056 Basel, Switzerland

**Keywords:** germanium, nanowires, silicon, CVD, VLS

## Abstract

The broad and fascinating properties of nanowires and their synthesis have attracted great attention as building blocks for functional devices at the nanoscale. Silicon and germanium are highly interesting materials due to their compatibility with standard CMOS technology. Their combination provides optimal templates for quantum applications, for which nanowires need to be of high quality, with carefully designed dimensions, crystal phase, and orientation. In this work, we present a detailed study on the growth kinetics of silicon (length 0.1–1 μm, diameter 10–60 nm) and germanium (length 0.06–1 μm, diameter 10–500 nm) nanowires grown by chemical vapor deposition applying the vapour–liquid–solid growth method catalysed by gold. The effects of temperature, partial pressure of the precursor gas, and different carrier gases are analysed via scanning electron microscopy. Argon as carrier gas enhances the growth rate at higher temperatures (120 nm/min for Ar and 48 nm/min H${}_{2}$), while hydrogen enhances it at lower temperatures (35 nm/min for H${}_{2}$ and 22 nm/min for Ar) due to lower heat capacity. Both materials exhibit two growth regimes as a function of the temperature. The tapering rate is about ten times lower for silicon nanowires than for germanium ones. Finally, we identify the optimal conditions for nucleation in the nanowire growth process.

## 1. Introduction

Nanowires (NW) have gained a great deal of attention in the last decades due to their fascinating properties, e.g., large surface-to-volume ratio [1,2], quantum confinement of charge carriers [3,4], and high carrier mobility [5]. These properties, if controlled on demand, can be used to engineer building blocks of functional devices at the nanoscale, finding applications in, e.g., optoelectronics [6,7], electronics [6,8], and photovoltaics [9,10,11]. To this end, control of the dimensions, crystal orientation, crystal phase, and density of crystal defects is crucial. The electrical properties of graphene sheets [12], organic molecules [12], and semiconductor nanowires [13] have been studied; however, most of these systems are not compatible with complementary metal oxide semiconductor (CMOS) technology, and as such are less likely to be incorporated into well-established fabrication processes.

Group IV materials, in particular germanium (Ge) and silicon (Si), offer potential ease of integration for the microelectronics industry [7,14,15,16]. The structural and electrical properties of Si nanowires [2,11] and the intrinsic high carrier mobility of Ge nanowires [17,18,19,20,21] make the combination of these two materials a perfect template for photodetection [2,22,23], energy storage [24,25], and quantum computing [26,27,28]. In order to integrate nanowires into large-scale applications and complex architectures, a high level of control of the growth mechanism is needed.

Theoretical modelling and understanding of the change of properties when switching from the bulk to the nanometer scale is of great value for further optimisation of the growth process. Using first principles calculations has already shown good predictive results [29]. Different materials have been modelled, including Ge [30,31,32], Si [30,33,34], SiGe [30,35,36], ZnO [37,38], GaAs [39], and InN [40]. Despite the huge application potential of Ge and Si nanowires, no theoretical publications have yet shed light on the influence of their growth parameters. Recent research has focused mostly on investigating the properties of Ge and Si nanowires, with particular emphasis on their electrical [38,41,42,43,44], thermal [45,46], and optical properties [38,47,48].

Nanowires can be grown using a top-down or bottom-up approach [12,49,50,51]. Usually, bottom-up nanowires are grown via the so-called vapour–liquid–solid (VLS) approach [49,52,53,54,55], which was first reported in 1964 [56]. The VLS method offers the unique possibility of merging a large variety of material combinations, which is due to the strain relaxation mechanism in nanowires [57,58]. The effectiveness of VLS has been demonstrated for a wide range of combinations already [59,60,61,62,63,64]. In VLS, a metallic nanoparticle, e.g., gold, is used to catalyze growth. This nanoparticle forms a liquid eutectic droplet with the underlying substrate at the eutectic temperature. The precursor gas decomposes onto the surface of the liquid catalyst and starts to accumulate inside the liquid droplet upon supersaturation. At this point, the wires start to precipitate in a layer-by-layer manner at the interface between the catalyst and the substrate; the diameter of the nanowire is defined by the catalyst droplet size [65,66]. Different metals can be used as catalysts, with one of the most common for Si and Ge nanowires being gold. The catalyst droplet can be obtained either as the result of dewetting a thin film [67,68], as a lithographically fabricated nanoparticle array [69,70], or by depositing a colloidal solution on the substrate [54,71,72]. Gold has proven to be an ideal catalyst for hydride precursors; it is redox-inactive [73], exhibits a relatively low eutectic temperature, and has many key semiconducting materials, including silicon and germanium. For Si and Ge in combination with Au, the eutectic temperature is around 360 °C [74,75,76,77,78,79].

Different techniques can be used to grow NWs, mainly differing in the way that the precursor is provided to the catalyst, with examples including metal–organic chemical vapour deposition, molecular beam epitaxy, and chemical vapour deposition (CVD). CVD is a commonly used technique to grow nanowires [6,18,51,68,80], offering epitaxial growth along with the required control over size, composition, and morphology. In order to grow high quality nanowires, it is vital to understand the influence of the growth conditions. Because CVD offers a large parameter space in terms of growth conditions, a thorough investigation of their impact on nanowire morphology and crystal properties is necessary. In this article, we present a systematic study of Si and Ge nanowire growth conditions. The wires were grown onto an Si <100> substrate via VLS using gold as catalyst. The effects of temperature and partial pressure were evaluated, and the influence of carrier gases was considered. To the best of our knowledge, no previous studies have provided these insights into the effects of carrier gases on the growth of nanowires using these materials.

## 2. Materials and Methods

The nanowires (NWs) used in this study were all grown using the VLS approach under a gold-catalysed reaction. The sample preparation prior to growth can be described in two steps. In the first step, gold nanoparticles with a diameter of 5 nm were deposited onto an undoped Si <100> substrate (float zone, undoped, resistivity >10,000Ohmcm, UniversityWafer Inc., South Boston, MA, USA) from a commercial colloidal suspension (SPI Supplies and BBI Solutions), which was diluted in deionized water in a ratio of 1:10. An electrostatic approach was employed for deposition, which was triggered by the addition of HCl 0.1 M [81]. In the second step, the samples were immersed in a 2% HF aqueous solution for 1 min to remove the native silicon oxide, with a load time after HF etching of no longer than 8 min. Afterwards, the NWs were grown using a PlasmaPro 100 Nanofab reactor from Oxford Instruments, Wiesbaden, Germany (base pressure <0.5mTorr). As the precursor gases, we used commercially available germane gas (${\mathrm{GeH}}_{4}$, PanGas AG, Switzerland, 99.999%) and silane gas (${\mathrm{SiH}}_{4}$, PanGas AG, Dagmersellen, Switzerland, 99.999%), while we used argon and hydrogen (Ar, $H_{2}$, PanGas AG, Switzerland, 99.999%) as the dilution carrier gases.

The growth protocol consisted of three steps: (i) preheating the chamber to the growth temperature; (ii) loading the sample immediately after HF; and (iii) introduction of the reaction gas mixture, consisting of the precursor gas and carrier gas, subsequent to the loading of the sample. Upon inlet of the gas mixture, the pressure increased to the preset pressure, which depended on the experiment being conducted.

**Figure 1:**
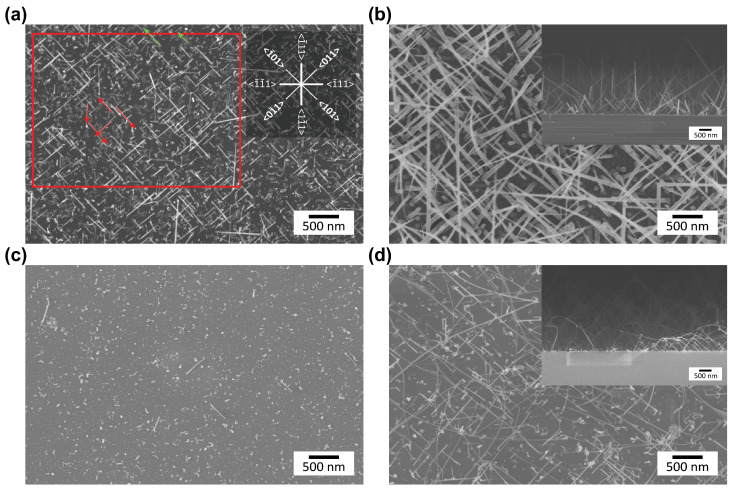
Typical SEM images of Ge and Si NWs grown under different conditions in an Ar atmosphere. The images were used to evaluate the growth rate, crystal direction, and nucleation rate. (**a**) Ge NWs grown at 350 °C and 200 mTorr. Exemplary arrows indicate the growth direction of the NWs and which wires were taken into account (green and red arrows). The crystal orientation can be inferred from the angle of the line. The wind rose (top right) shows the main and statistically more probable growth directions. (**b**) Ge NWs grown at 350 °C and 1 Torr, including a cross-sectional view of the sample in the inset. (**c**) Si NWs grown at 450 °C and 1 Torr. (**d**) Si NWs grown at 600 °C and 1 Torr, including a cross-sectional view of the sample in the inset.

After growth, the NWs were imaged using scanning electron microscopy (SEM) to determine the NW dimensions and orientation. Therefore, the SEM scan direction was oriented parallel to the natural cleaving plane of the Si chip, with the edge lines following ⟨110⟩ directions. Figure 1 shows a top view SEM image of the resulting NWs. We have added an inset in the top right corner of Figure 1 indicating the ⟨110⟩ directions as diagonal lines and the ⟨111⟩ directions as horizontal and vertical lines.

For statistical evaluation of the obtained wires, we defined a protocol with which we analysed three images from different areas of each as-grown sample, which were about 1 to 2 mm apart from each other. We predefined an area (the red rectangle in Figure 1) and considered only those NWs for which growth started inside this area. We manually drew lines according to the growth direction of the wires, taking into account those wires fully contained within the predefined area (the red arrows in Figure 1) and those ending outside the predefined area (the green arrows in Figure 1). Unless otherwise specified, all possible growth directions of the NWs were considered in our statistical analysis of the influence of the growth parameters, although only the main and statistically more probable growth directions (<110> and <111>) are indicated in the inset of Figure 1**a**. Crossing of NWs was been considered in the statistics according to their base directions. In this way, both the length of the NWs and their orientation was determined while taking into account the length correction due to the projection of the angle of view. We used ImageJ software (Version 1.48s), and the scale was set automatically according to the SEM image. Growth rates were calculated by averaging the individual length of the NWs for each sample and dividing the result by the growth time without differentiating between growth directions.

For the nucleation maps, only the angles representing the <110> (45±10°) and <111> (0+5° and 90−5°) growth directions were considered. The colloid density used to calculate the nucleation rates was determined by SEM images of the exemplary samples after deposition of the colloids.

## 3. Results and Discussion

In order to investigate the influence of partial pressure and temperature on the growth rate and tapering rate of Ge and Si NWs, different growth conditions were evaluated by means of SEM imaging techniques.

**Figure 2:**
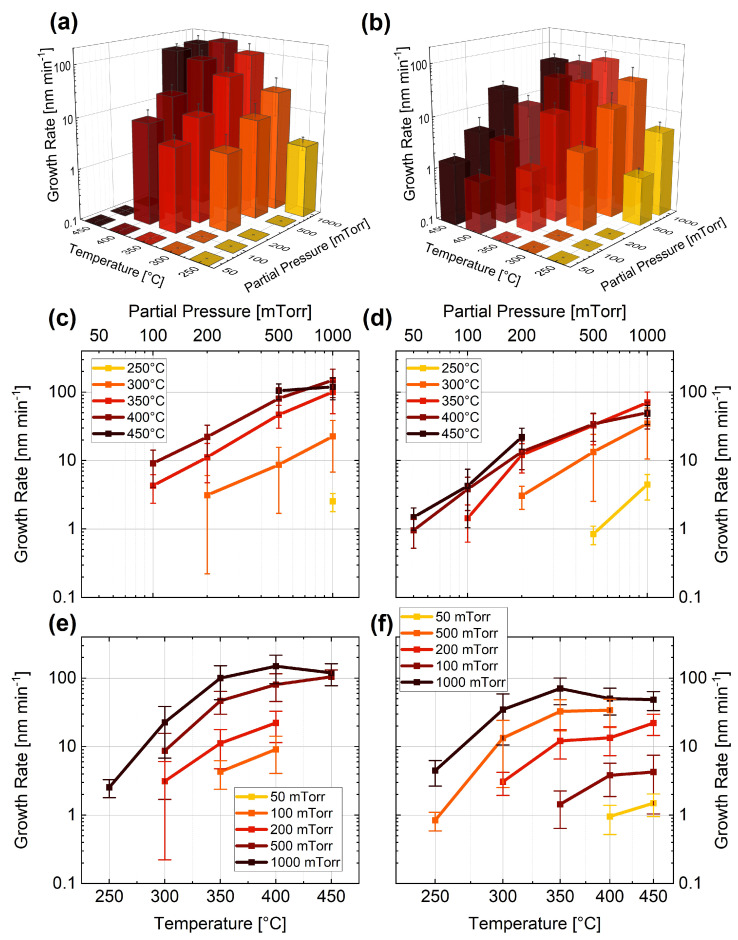
Dependence of the growth rate of Ge NWs on ${\mathrm{GeH}}_{4}$ partial pressure and temperature for (**a**,**c**,**e**) 10%${\mathrm{GeH}}_{4}$ in Ar and (**b**,**d**,**f**) 10%${\mathrm{GeH}}_{4}$ in $H_{2}$.

In Figure 2, the dependence of the growth rate on the temperature and partial pressure of the precursor gas is presented for 10%${\mathrm{GeH}}_{4}$ in Ar (**a**,**c**,**e**) and 10%${\mathrm{GeH}}_{4}$ in $H_{2}$ (**b**,**d**,**f**). Qualitatively, a linear behaviour of the growth rate for increasing partial pressure can be observed for both carrier gases (Ar and $H_{2}$ in Figure 2**a** and Figure 2**b**, respectively). This observation is further highlighted in Figure 2**c**,**d**, and is related to a higher rate of Ge being incorporated into the liquid eutectic when the pressure is increased [82]. Interestingly, no growth could be observed when setting a growth temperature of 450 °C and a partial pressure of 500 mTorr using $H_{2}$ as a precursor gas. While the reason for this is not yet clear, this result was reproduced multiple times.

By comparing the results obtained with the two carrier gases, another trend can be identified in Figure 2**a**,**b**. When $H_{2}$ is used as carrier gas, the growth rate is reduced for higher temperatures (e.g., 48±15 nm/min in $H_{2}$ compared to 120±43 nm/min in Ar at 450 °C and 1 Torr) and enhanced for lower temperatures (e.g., 35±24 nm/min in $H_{2}$ compared to 22±15 nm/min in Ar at 300 °C and 1 Torr). The high-temperature behaviour can be explained by the lower specific heat capacity of $H_{2}$, which results in slower dissociation of ${\mathrm{GeH}}_{4}$ and consequently in a reduced growth rate [83]. The low-temperature regime (below 350 °C) investigated in this study is below the desorption temperature of $H_{2}$ [84,85], resulting in a more dominant enhancement of the growth rate. The overall observed growth range is enhanced with $H_{2}$, especially for low partial pressures and high-temperature regions (above 350 °C).

**Figure 3:**
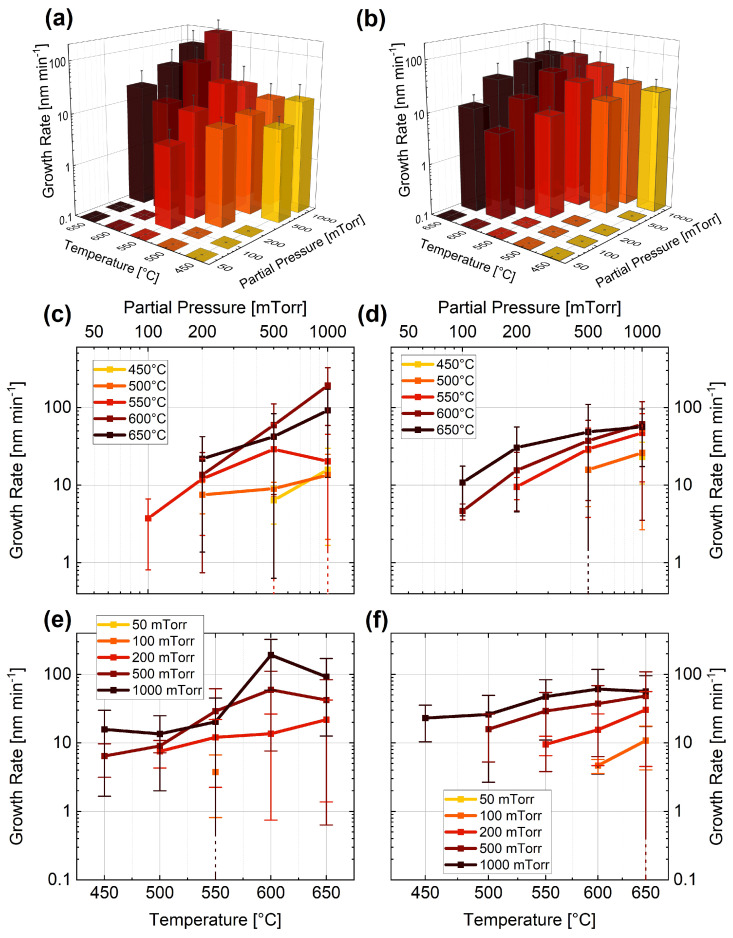
Growth rate (**a**–**f**) dependence of Si NWs on ${\mathrm{SiH}}_{4}$ partial pressure and temperature for (**a**,**c**,**e**) 10%${\mathrm{SiH}}_{4}$ in Ar and (**b**,**d**,**f**) 10%${\mathrm{SiH}}_{4}$ in $H_{2}$. Note that the dashed error bars account for the standard deviation of the dataset; the end of the error bar is not visible within the displayed range.

The growth rate for Si NWs and their dependence on the partial pressure of ${\mathrm{SiH}}_{4}$, temperature, and carrier gas are presented in Figure 3. As gas mixtures, we used 10%${\mathrm{SiH}}_{4}$ in Ar (**a**,**c**,**e**) and 10%${\mathrm{SiH}}_{4}$ in $H_{2}$ (**b**,**d**,**f**). Similar to the case of the Ge NWs, qualitatively, a linear dependence of the growth rate on pressure is observed for Si NWs (see Figure 3**c**,**d**). This dependence is due to a higher incorporation rate of Si into the liquid eutectic [82] at higher pressures. On the other hand, we observed an exponential dependence on temperature when using Ar as the carrier gas and a linear dependence when using $H_{2}$ (see Figure 3**e** and Figure 3**f**, respectively). This different dependence can be further explained by the larger enhancement of the growth rate with Ar due to its higher specific heat capacity [83].

**Figure 4:**
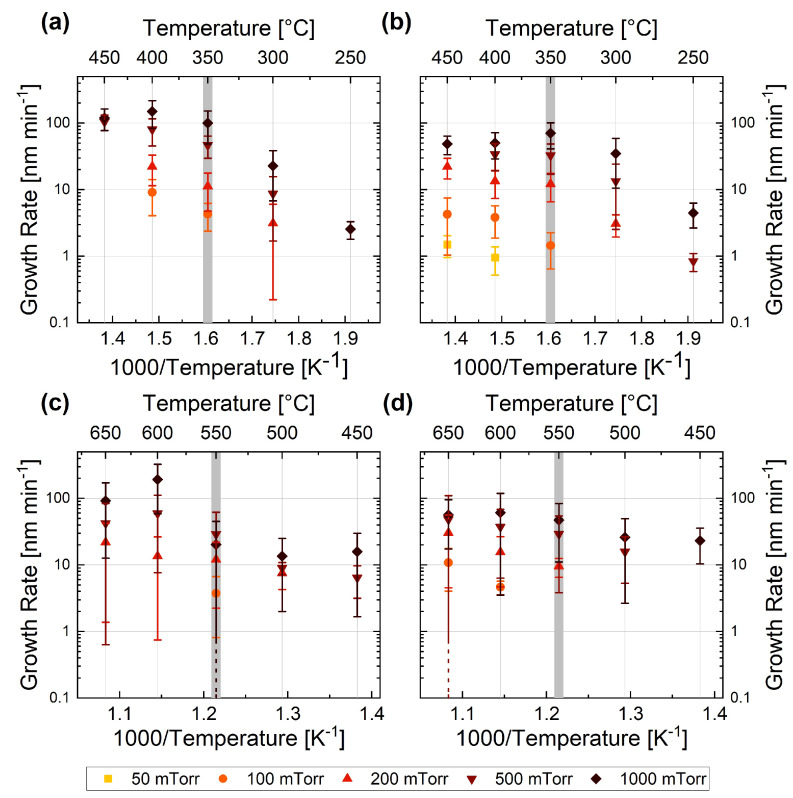
Arrhenius plots for different precursors and carrier gases: (**a**) 10%${\mathrm{GeH}}_{4}$ in Ar, (**b**) 10%${\mathrm{GeH}}_{4}$ in $H_{2}$, (**c**) 10%${\mathrm{SiH}}_{4}$ in Ar, and (**d**) 10%${\mathrm{SiH}}_{4}$ in $H_{2}$. In each plot, the transition between the two growth regimes is indicated by the light grey line. Note that the dashed error bars account for the standard deviation of the dataset; the end of the error bar is not visible within the displayed range.

For both material systems, two different temperature-dependent growth regimes can be identified for higher pressures (Figure 2**c**,**d** and Figure 3**c**,**d**), as is further discussed below and highlighted in Figure 4.

In the Arrhenius plots in Figure 4, the growth rate is plotted against the inverse of the temperature. Independent of the type of carrier gas, two different growth rate regimes can be observed for high-pressure conditions (partial pressure > 200 mTorr), as highlighted by the light grey line in panels (**a**) to (**d**), which indicates the transition temperature. The transition temperatures between the two regimes are at around 350 °C for Ge NWs and 550 °C for Si NWs in both Ar and $H_{2}$, which is in good agreement with previously reported experiments [86,87]. These transition temperatures were ascertained using a quantitative method, for which two linear fits in the intervals of (350 °C, N) and (N, 450 °C) for Ge NWs and (450 °C, N) and (N, 650 °C) for Si NWs were computed, yielding the residual of squares. We linearly fitted the logarithm of the growth rates (data plotted in Figure 4) with the Arrhenius equation provided by

(1) $$\ln\left(\mathrm{growth}\;\mathrm{rate}\right)=\frac{-E_{A}}{R}\cdot \frac{1}{T},$$

where R is the universal gas constant and $E_{A}$ is the activation energy. The calculated activation energies for the different regimes, precursors, and carrier gases are presented in Table 1.

For the Ge NWs, a lower activation energy can be observed for lower temperature (below 350 °C) and a higher one for higher temperature (above 350 °C). The increase in activation energy at higher temperatures can be related to a competing effect between the temperature-dependent decomposition of the precursor gas and the uncatalysed deposition on the side facets and the substrate, resulting in a higher tapering rate, as identified in Figure 2 [88]. The activation energy values of around $90\;\mathrm{kJ}\cdot {\mathrm{mol}}^{-1}$ estimated for ${\mathrm{GeH}}_{4}$ for temperatures above 350 °C (the high temperature in Table 1) are in good agreement with the values reported in the literature [89,90]. The values for the low-temperature region (the low temperature in Table 1) are three to six times smaller compared to the value of $138.07\;\mathrm{kJ}\cdot {\mathrm{mol}}^{-1}$ reported for thin film deposition in [86]. This reduction in activation energy is consistent with a catalysed reaction; therefore, it is coherent with the VLS mechanism leading to NW growth.

Considering the values of the activation energy, the estimate for ${\mathrm{SiH}}_{4}$ is about half the value reported in the literature for similar temperature regions, ranging from 89 to $230\;\mathrm{kJ}\cdot {\mathrm{mol}}^{-1}$ below 550 °C (the low temperature in Table 1) [12,75,87,91,92] and 74 to $89\;\mathrm{kJ}\cdot {\mathrm{mol}}^{-1}$ above 550 °C (the high temperature in Table 1) [74,93]. However, our results are consistent with the trends observed previously, i.e., higher activation energy for lower temperatures and lower activation energy for higher temperatures. The catalytic effect of Au may be related to the lower activation energy for the lower temperatures compared to the thin film deposition reported in literature, ranging from 125 to $214\;\mathrm{kJ}\cdot {\mathrm{mol}}^{-1}$ [87,91]. Furthermore, it has previously been observed that the activation energy depends on the size of the catalyst [75]. In our study, we used Au particles with a small size (nominal colloid size of 5 nm), further increasing the consistency of our values for the activation energy compared to those reported in literature.

**Figure 5:**
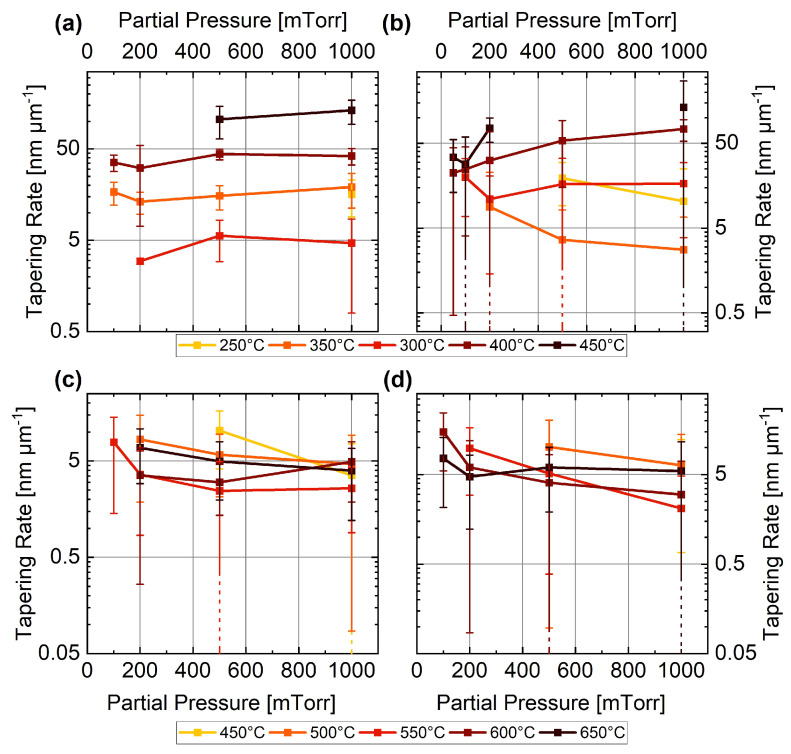
Dependence of the tapering rate on the temperature for (**a**) 10%${\mathrm{GeH}}_{4}$ in Ar, (**b**) 10%${\mathrm{GeH}}_{4}$ in $H_{2}$, (**c**) 10%${\mathrm{SiH}}_{4}$ in Ar, and (**d**) 10%${\mathrm{SiH}}_{4}$ in $H_{2}$. Note that the dashed error bars account for the standard deviation of the dataset; the end of the error bar is not visible within the displayed range.

The tapering rates for Ge and Si NWs and their dependence on the partial pressure of ${\mathrm{GeH}}_{4}$ and ${\mathrm{SiH}}_{4}$ are presented in Figure 5. We used 10% precursor gas (${\mathrm{GeH}}_{4}$ and ${\mathrm{SiH}}_{4}$) in Ar (**a**,**b**) and $H_{2}$ (**c**,**d**) as gas mixtures. Increasing the partial pressure did not have an effect on the tapering rate independent of the type of carrier gas; instead, we observed a dependence of the tapering rate on the temperature, especially for the Ge NWs. This dependence can be explained by the fact that uncatalysed decomposition is less dependent on the pressure, and is mainly dependent on the temperature [82].

**Figure 6:**
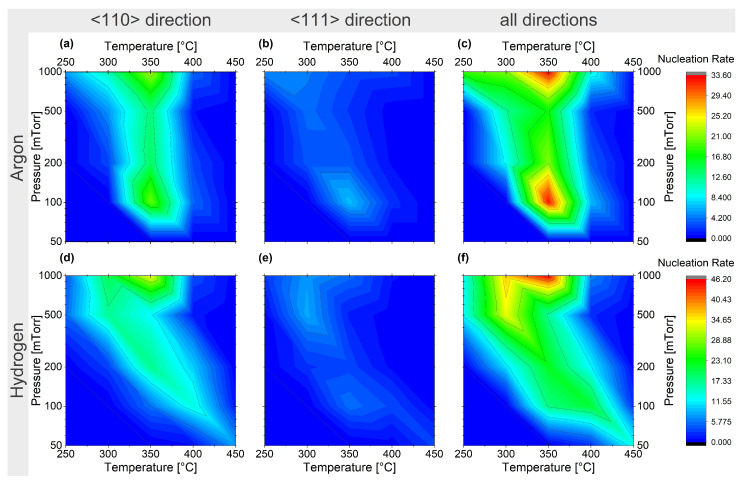
Nucleation map of Ge NWs from Au catalysts for 10%${\mathrm{GeH}}_{4}$ in Ar (**a**–**c**) and 10%${\mathrm{GeH}}_{4}$ in $H_{2}$ (**d**–**f**) for the growth directions <110> (**a**,**d**), <111> (**b**,**e**), and all observed wires (**c**,**f**).

Another important aspect of the growth of NWs is the possibility of controlling the growth direction. Figure 6 shows the nucleation maps for Ge NWs, providing an overview of the growth conditions. A high yield of NWs can be expected along specific growth directions. When using Ar as the carrier gas, Figure 6**a**–**c** shows a clear preferred nucleation temperature around 350 °C, including two favourable growth conditions at 1000 mTorr and 100 mTorr partial pressure. The observed temperature of 350 °C corresponds well with the eutectic temperature for Si and Au [86,94]. A similar preferred temperature can be observed when using $H_{2}$ as the carrier gas (Figure 6**c**–**f**), although in this case there is only one growth condition favourable to high nucleation at around 1000 mTorr partial pressure, which corresponds well with the lower specific heat of $H_{2}$. This is consistent with previously reported experimental findings showing enhanced growth with Ar compared to $H_{2}$ [90]. The larger nucleation region for the high-pressure regime is related to the higher probability of the precursor gas reacting on the surface of the Au catalyst compared to lower pressures [82]. Another interesting observation is the decrease in nucleation for temperatures above 400 °C. Here, the catalyzed nucleation from the gold particles must compete with the growth of thin films on the substrate, which bury the gold particles underneath and inhibit the growth effect caused by the gold.

**Figure 7:**
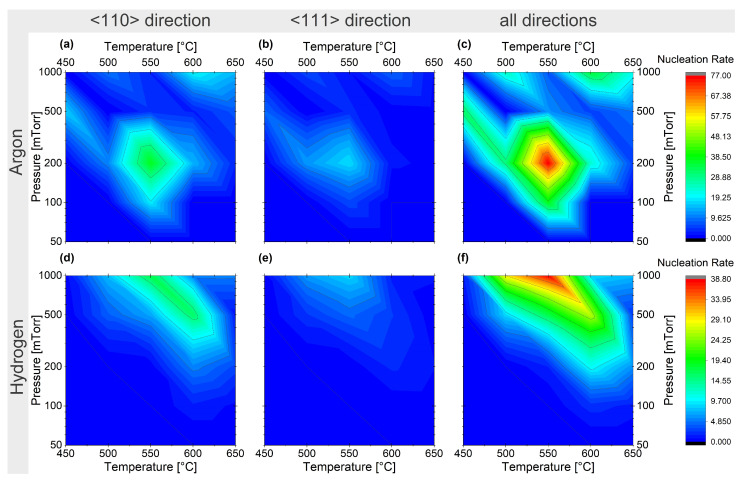
Nucleation map of Si NWs from Au catalysts for 10%${\mathrm{SiH}}_{4}$ in Ar (**a**–**c**) and 10%${\mathrm{SiH}}_{4}$ in $H_{2}$ (**d**–**f**) for the growth directions <110> (**a**,**d**), <111> (**b**,**e**), and all the observed wires (**c**,**f**).

We display the nucleation maps of Si NWs for Ar and $H_{2}$ as carrier gases in Figure 7. Clearly favourable growth conditions for Si NWs grown in Ar atmosphere can be identified at around 550 °C and 200 mTorr partial pressure (**c**), and the effect is stronger for the <110> growth direction (**a**) than for the <111> direction (**b**). These results can be related to the higher binding energy of ${\mathrm{SiH}}_{4}$ [95,96]. Using $H_{2}$ as the carrier gas shifts the favourable growth conditions to higher pressures (Figure 7**d**,**e**). This behaviour can be explained by the higher specific heat of Ar, which leads to a warmer environment due to less heat transport away from the sample.

Comparing the probability of obtaining Ge and Si NWs grown along the <110> and the <111> growth directions (Figure 6 and Figure 7), the <110> growth direction shows a nucleation rate two to three times higher for the favourable growth conditions established above; this results from minimization of the surface free energy, which strongly influences the growth direction [97] due to the small diameter of the colloids used in this study.

## 4. Conclusions

We carried out a study on the growth kinetics of Ge and Si nanowires for different precursor gases, temperatures, and partial pressures. The resulting data showed the expected linear behaviour when increasing the partial pressure, along with two different growth regimes as a function of temperature. The transition temperatures were 350 °C and 550 °C for and Ge and Si, respectively. A general enhancement of the growth rate was observed when using Ar as carrier gas compared to $H_{2}$. Our results demonstrate that the tapering rate is almost constant as a function of the partial pressure, and as such is more sensitive to temperature variations. In particular, Si NWs exhibited low tapering, while Ge NWs exhibited a non-negligible increase in tapering with the temperature for both carrier gases. Favourable nucleation growth conditions were identified for the different precursor and carrier gases, showing a higher probability for the <110> growth direction.

## Figures and Tables

**Table 1:** Extracted values for the activation energies of the high- and low-temperature regimes for the different carrier and precursor gases.

Gas Mixture	Low Temperature [$\mathrm{kJ}\cdot {\mathrm{mol}}^{-1}$]	High Temperature [$\mathrm{kJ}\cdot {\mathrm{mol}}^{-1}$]
10% ${\mathrm{GeH}}_{4}$ in Ar	34.38±20.60	93.06±14.90
10% ${\mathrm{GeH}}_{4}$ in $H_{2}$	19.88±26.33	90.21±15.04
10% ${\mathrm{SiH}}_{4}$ in Ar	57.11±46.59	34.00±21.59
10% ${\mathrm{SiH}}_{4}$ in $H_{2}$	56.42±44.51	48.44±22.86

## Data Availability

The data presented in this study are available on request from the corresponding author.

## Abbreviations

The following abbreviations are used in this manuscript:

Ar	Argon
CMOS	Complementary Metal Oxide Semiconductor
CVD	Chemical Vapour Deposition
Ge	Germanium
${\mathrm{GeH}}_{4}$	Germane
$H_{2}$	Hydrogen
NW	Nanowire
SEM	Scanning Electron Microscope
${\mathrm{SiH}}_{4}$	Silane
Si	Silicon
VLS	Vapour–Liquid–Solid
